# Effective-compound combination of Bufei Yishen formula III combined with ER suppress airway mucus hypersecretion in COPD rats: via EGFR/MAPK signaling

**DOI:** 10.1042/BSR20222669

**Published:** 2023-11-10

**Authors:** Kexin Xu, Jindi Ma, Ruilong Lu, Xuejie Shao, Yakun Zhao, Lili Cui, Zhiguang Qiu, Yange Tian, Jiansheng Li

**Affiliations:** 1 Henan Key Laboratory of Chinese Medicine for Respiratory Disease, Co-construction Collaborative Innovation Center for Chinese Medicine and Respiratory Diseases by Henan and Education Ministry of P.R., Henan University of Chinese Medicine, Zhengzhou, Henan 450046, China; 2 Traditional Chinese Medicine (ZHONG JING) School, Henan University of Chines Medicine, Zhengzhou, Henan 450046, China; 3 Institute for Respiratory Diseases, The First Affiliated Hospital, Henan University of Traditional Chinese Medicine, Zhengzhou, Henan 450008, China

**Keywords:** Airway mucus hypersecretion, Chinese medicine, Chronic obstructive pulmonary disease, Exercise Rehabilitation

## Abstract

Background: The aim of this study was to explore the combined efficacy ofeffective-component compatibility of Bufei Yishen formula III (ECC-BYF III) and exercise rehabilitation (ER) in inhibiting airway mucus hypersecretion in a chronic obstructive pulmonary disease (COPD) rat model.

Methods: A total of 48 SD rats were divided into control, model, acetylcysteine (NAC), ECC-BYF III, ER, and ECC-BYF III + ER groups (*n*=8). COPD rats were exposed to cigarette smoke and bacteria for 8 weeks and administered various treatments over the next eight weeks. Rats were euthanized at week 17 after pulmonary function testing. Pathological examination of lung tissues was performed. IL-6 and IL-10 levels were measured in bronchoalveolar lavage fluid (BALF) and protein levels of MUC5AC, MUC5B, AQP-5, EGFR, ERK, JNK, and p38 were measured in lung tissues.

Results: Improved pulmonary function and pathological changes were observed in ECC-BYF III, ECC-BYF III + ER, and NAC groups. ECC-BYF III and ECC-BYF III + ER had greater mean alveolar number (MAN) compared with NAC. Lung inflammation and goblet cell generation were reduced and MUC5AC, MUC5B and AQP-5 expressions were lower in all treatment groups. ECC-BYF III has more significant effect on MUC5AC than ER and NAC. ECC-BYFIII + ER had a greater effect on suppressing IL-6 in BALF compared with other treatments. ECC-BYFIII, ER, and ECC-BYF III + ER reduced EGFR, ERK, JNK, and p38 phosphorylated protein levels. ECC-BYFIII+ER had a greater effect on p-JNK and p-p38 than ECC-BYFIII and NAC.

Conclusion: ECC-BYF III, ER, and ECC-BYF III + ER have efficacy in inhibiting airway mucus hypersecretion with improved pulmonary function and pathological changes. ECC-BYF III had a greater effect in improving MAN and MUC5AC in lung tissue. ECC-BYF III+ER had a greater effect in alleviating pulmonary pathology and inflammation. These effects may be mediated by inhibition of the EGFR/MAPK pathway.

## Introduction

**Figure 1:**
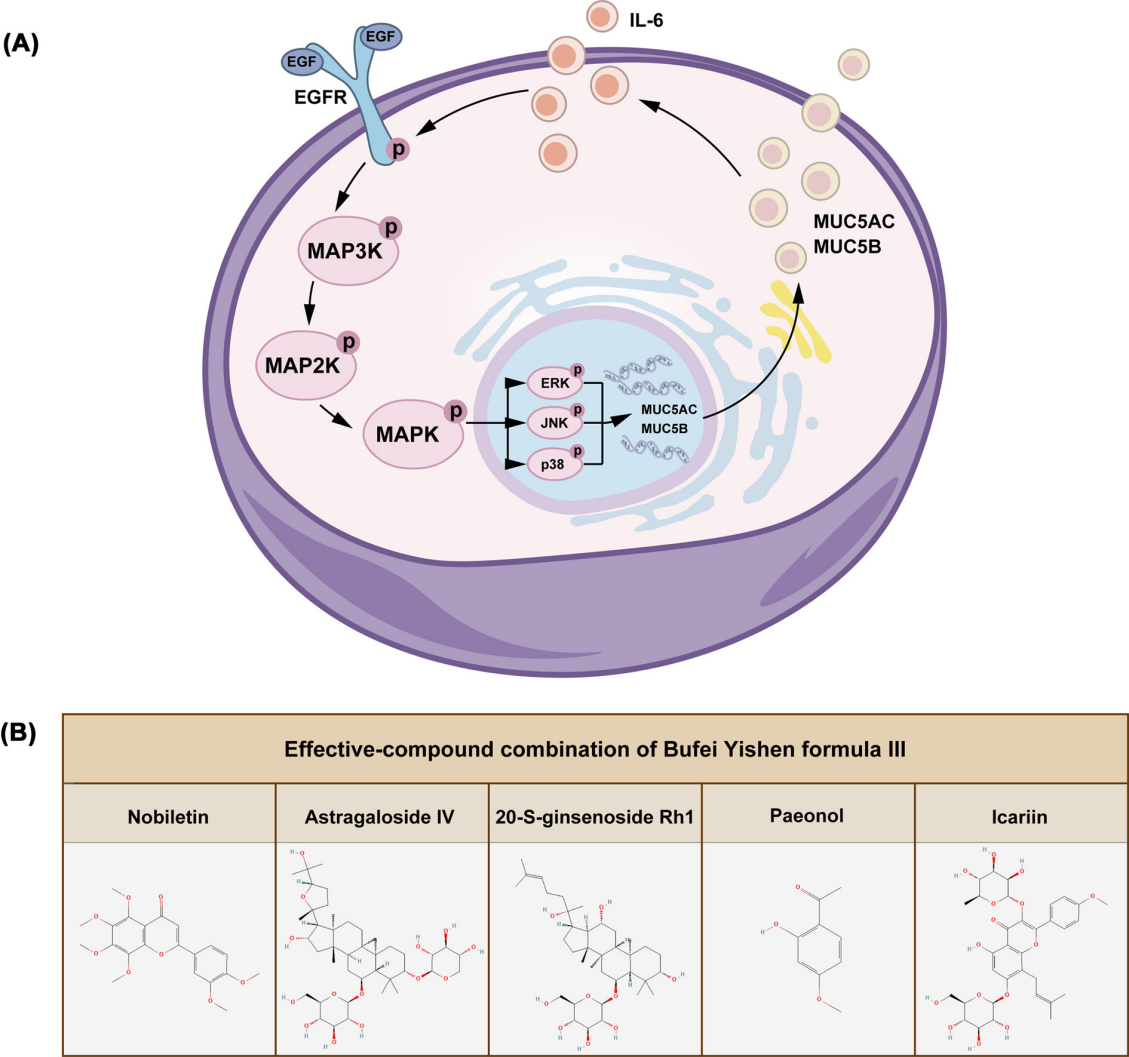
EGFR/MAPK mechanism and chemical structures of ECC-BYF III (**A**) Schematic diagram of EGFR/MAPK. (**B**) The chemical structure of the five compounds in ECC-BYF III.

Chronic obstructive pulmonary disease (COPD) is characterized by persistent respiratory symptoms and airflow limitation [1] and represents a serious hazard to public health. Accordingly, there is an urgent need for novel clinical treatments due to the high morbidity and mortality of COPD [2]. Airway mucus hypersecretion (AMH) is an independent risk factor and contributes to the pathophysiology of COPD. COPD is primarily caused by an increase in goblet cells and the enlargement of submucosal glands. Oxidative stress and inflammation, which are caused by cigarette smoke (CS) and harmful substances, lead to airway epithelial injury, mucus hypersecretion, thereby leading to chronic cough and bronchitis. Excessive mucus blockage of airways can lead to airflow restriction and dyspnea, further exacerbating COPD [3,4]. Therefore, the inhibition of mucus hypersecretion and the promotion of effective sputum excretion represent effective strategies for reducing acute exacerbations of COPD and delaying disease progression. EGFR/MAPK signaling is involved in regulating airway mucus secretion and increasing airway goblet cell hyperplasia and mucus hypersecretion [5]. The synthesis of MUC5AC is regulated by the EGFR signaling pathway, with the EGFR pathway known to be activated by direct damage to airway epithelial cells by oxidants and arachidonic acid metabolites in smoke [6]. A variety of ligands have been shown to bind to EGFR, promote EGFR phosphorylation, and activate multiple downstream pathways including the MAPK pathway with corresponding biological effects. ERK, JNK, and p38, as the key mediators of the MAPK pathway, are closely related to AMH (Figure 1A) [7]. Accordingly, inhibition of the EGFR/MAPK pathway represents a major therapeutic target for inhibiting mucus hypersecretion.

Mucolytics are the most important drugs used to manage AMH and its sequelae including COPD and bronchiectasis and function by enhancing the clearance of the mucus layer of the respiratory tract [1,8]. Mucolytics including N-acetylcysteine, carbocysteine, erdosteine, and dornase alfa have antioxidative and anti-inflammatory properties in addition to the mucolytic action described above. However, many mucolytics are associated with adverse effects such as vomiting, diarrhea, laryngitis, gastric ulceration, and chest pain. Further, overdose of mucolytics can lead to thrombocytopenia, hemolysis, and acute renal failure [4]. In recent years, Traditional Chinese Medicine (TCM) is playing an increasingly important role in treating AMH and COPD due to efficacy in alleviating symptoms, reducing the frequency of acute exacerbations frequency, and improving overall health [9,10]. Our previous study demonstrated that Bufei Yishen formula (BYF; patent ZL.201110117578.1) can restore protease/antiprotease and reduce oxidative stress and airway inflammation, thereby improving the clinical symptoms and quality of life of patients with COPD and reducing the number of acute exacerbations [11,12]. Effective-component compatibility of Bufei Yishen formula I (ECC-BYF I) and effective-component compatibility of ECC-BYF II (ECC-BYF II) were developed following network pharmacologic analysis of the active components of BYF, *in vitro* experiments involving multiple cell models, and *in vivo* verification and optimization [13,14]. ECC-BYF III was obtained by further optimization with the addition of astragaloside IV and 20-S-ginsenoside Rh1 among other components. ECC-BYF III has a simple composition with same efficacy and safety as BYF [15]. ECC-BYF III has been shown to have efficacy in a rat model of COPD by enhancing pulmonary function, reducing oxidative stress, inflammation, and mucin secretion [15,16]; however, the mechanisms underlying the effects of ECC-BYF III on AMH and COPD remains unclear.

Pulmonary rehabilitation exercise and acupuncture have achieved good results in clinical studies of COPD [17,18]. Exercise rehabilitation (ER) represents an effective means of disease treatment and rehabilitation and is an important component of pulmonary rehabilitation programs [1]. Proper exercise can effectively improve pulmonary function and respiratory muscle endurance in patients with COPD, thereby improving dyspnea and exercise tolerance and reducing hospital admissions and mortality [19,20]; however, the mechanisms underlying the effects of ER remain unclear. Herein, we evaluated the efficacy of ECC-BYF III and ER in inhibiting AMH in a rat model of COPD and evaluated the mechanisms underlying these effects with a particular focus on the EGFR/MAPK signaling pathway.

## Materials and methods

### Animal husbandry

Sprague–Dawley (SD) rats aged 6–7 weeks and weighing 220 ± 20 g were procured from Beijing Vital River Laboratory Animal Technology Co., Ltd (Special Pathogen Free, No. 110011211105823815). All animal experiments took place at Henan University of Chinese Medicine. The procedures of the present study were approved by the Experimental Animal Care and Ethics Committees of the First Affiliated Hospital of Henan University of Chinese Medicine with the ethical review approval number YFYDW2019031. Rats were killed by intraperitoneal injection of 2% pentobarbital sodium at 40 mg/kg.

### Drug preparation

ECC-BYF III is composed of astragaloside IV and 20-S-ginsenoside Rh1, etc. (Chengdu Must BioTech Co., Ltd., Chengdu, China) (Figure 1B). The purity of these compounds was verified to be more than 98% by high-performance liquid chromatography. The acetylcysteine (NAC) is a kind of effective mucolytic recommended by Global Initiative for Chronic Obstructive Lung Disease (GOLD). So, we chose NAC (Zambon S.p.A, Italia; 600 mg/tablet) as the control drug.

### Establishment of a rat model of COPD

A total of 48 SD rats were housed under standardized environment conditions and given food and water *ad libitum* for 7 days, then were randomly assigned to six experimental groups as follows: normal control group, disease model group, ECC-BYF III group, ER group, ECC-BYF III + ER group, and the NAC group (*n*=8 for each). The COPD rat model was established by exposing rats to CS (Hongqi Canal® filter cigarettes; Henan Tobacco Industry, Zhengzhou, China) and Klebsiella pneumoniae (KP) (0.1 ml, 6 × 10^8^ CFU/ml; ID: 46,117-5a1; National Centre for Medical Culture Collection, Beijing, China) [21]. From weeks 1 to 8, SD rats were exposed to CS at a concentration of 3000 ± 500 ppm for 40 min twice a day in all groups except the control group. KP solution was dropped into alternating nostrils once every 5 days. Normal saline was used instead of bacterial solution in the normal control group.

From weeks 9 to 16, rats in the normal control and disease model groups were intragastrically administered 10 ml/kg sodium carboxymethyl cellulose. Rats in the NAC group were administered 54 mg/kg/d NAC suspension. Rats in the ECC-BYF III group were administered 5.5 mg/kg/d ECC-BYF III. Rats in the ER group were provided exercise by running at 8 m per min on an experimental treadmill for 20 min per day. The experimental schema was validated in a preliminary experiment (Supplementary Material S1). Rats in the ECC-BYF III + ER group were treated with ECC-BYF III combined with exercise using the same drug dosage and exercise regimen as provided to the ECC-BYF III and ER groups, respectively. The COPD rat model was evaluated according to symptoms, lung function, and pulmonary pathology.

Dosages of NAC were calculated using the following formula [22,23]:

$${\text{D}}_{\text{rat}}={\text{D}}_{\text{human}}\times ({\text{K}}_{\text{rat}}/{\text{K}}_{\text{human}})\times {\left({\text{W}}_{\text{rat}}\times {\text{W}}_{\text{human}}\right)}^{2/3}$$

Where *D* is the dose and *K* is the body shape index defined as *K* = *A*/*W*^2/3^ where *A* is the surface area in m^2^ and *W* is the weight in kg.

At week 17, rats were killed by intraperitoneal injection of 2% pentobarbital sodium at 40 mg/kg.

### Pulmonary function tests

Peak expiratory flow (PEF) and expiratory flow at 50% tidal volume (EF50) were measured every 4 weeks from week 0 to week 16 using whole-body plethysmography (Buxco, NC, U.S.A.).

### Lung and bronchus tissue morphology

Lung tissues were cut into slices of 4 mm thickness and fixed with 4% paraformaldehyde with conventional dehydration and embedding in paraffin. Sections were stained with hematoxylin and eosin or Alcian Blue/periodic acid-Schiff (AB-PAS). Images were taken using an optical microscope (Olympus, Japan) and used to calculate the mean linear intercept (MLI), mean alveolar number (MAN) and airway wall thickness.

Under microscopy (×200), six visual field were taken in each slice, and the alveolar number and the linear intercept in a fixed area of visual field were measured. MAN (/mm^2^) = *N*_a_/A. *N*_a_ is the number of pulmonary alveoli in each visual field. *A* is the area of the visual field. Then, we made a cross(+) under the visual field and counted the number of alveolar septum on the cross. MLI (μm) = *L*/*N*s. *N*s is the number of alveolar septum. *L* is total length of the cross. Under microscopy (×200), three visual field which include airway (100–300 μm in diameter) were taken in each slice, and select three internal diameters and corresponding external diameters of the airways, respectively, subtract and average value, then divide by 2, and this means the airway wall thickness.

### Immunohistochemistry staining

Six slices of lung tissues in each group were immunostained with a MUC5AC polyclonal antibody (E-AB-40037, 1:200, Elabscience) or a MUC5B polyclonal antibody (E-AB-15988, 1:500, Elabscience) and imaged under a microscope at 200 times magnification. Integral optical density was determined using IPP 6.0 software.

### Immunofluorescence

Lung slices were incubated with an AQP-5 antibody (sc-74402, 1:1000, Santa Cruz Biotechnology) overnight at 4°C. Slices were then incubated with secondary antibody (Goat Anti-Mouse IgG (H + L) Fluor594-conjugated, S0005, 1:1000, Affinity) for 20 min and counterstained with DAPI for 10 min.

### Measurements of IL-6 and IL-10 in bronchoalveolar lavage fluid

For bronchoalveolar lavage, 3 ml of 4°C normal saline was injected into the left bronchus and then pumped back into a centrifuge tube. This procedure was repeated three times. Samples were centrifuged at 3500 rpm for 15 min to obtain the liquid supernatant before storage at −80°C.

IL-6 and IL-10 levels in bronchoalveolar lavage fluid (BALF) were detected using a commercial ELISA kit (BD Bioscience pharmingen, U.S.A.) and quantified according to the manufacturer’s protocol.

### Western blotting

Phosphorylated EGFR (p-EGFR), phosphorylated ERK (p-ERK), phosphorylated JNK (p-JNK), phosphorylated p38 (p-p38), ERK, JNK, and p38 were detected by Western blotting using p-EGFR (3777S, 1:1000, CST), p-ERK (28733-1-AP, 1:1000, Proteintech), p-JNK (4668S, 1:1000, CST), p-p38 (4511, 1:1000, CST), ERK (16443-1-AP, 1:1000, Proteintech), JNK (9252, 1:1000, CST), p38 (8690, 1:1000, CST), and GAPDH (10494-1-AP, 1:3000, Proteintech) antibodies. Denatured proteins were subjected to electrophoresis and transferred to polyvinylidene difluoride membranes. Membranes were blocked with 5% skim milk in 1× TBST. Membranes were incubated with primary antibody at 4°C overnight and then the corresponding secondary antibody. Protein bands were detected using enhanced chemiluminescence reagents.

### Statistical analysis

Data are presented as mean ± standard error. Data were analyzed using IBM SPSS 22.0 software. One-way analysis of variance with an appropriate *post hoc* test was used to compare data with a parametric distribution. If the variances were homogeneous, LSD method was performed. If the variances were inconsistent, Dunnett T3 test was performed. *P*-values <0.05 were considered statistically significant.

## Results

### ECC-BYF III and ER improve pulmonary function in COPD rats

**Figure 2:**
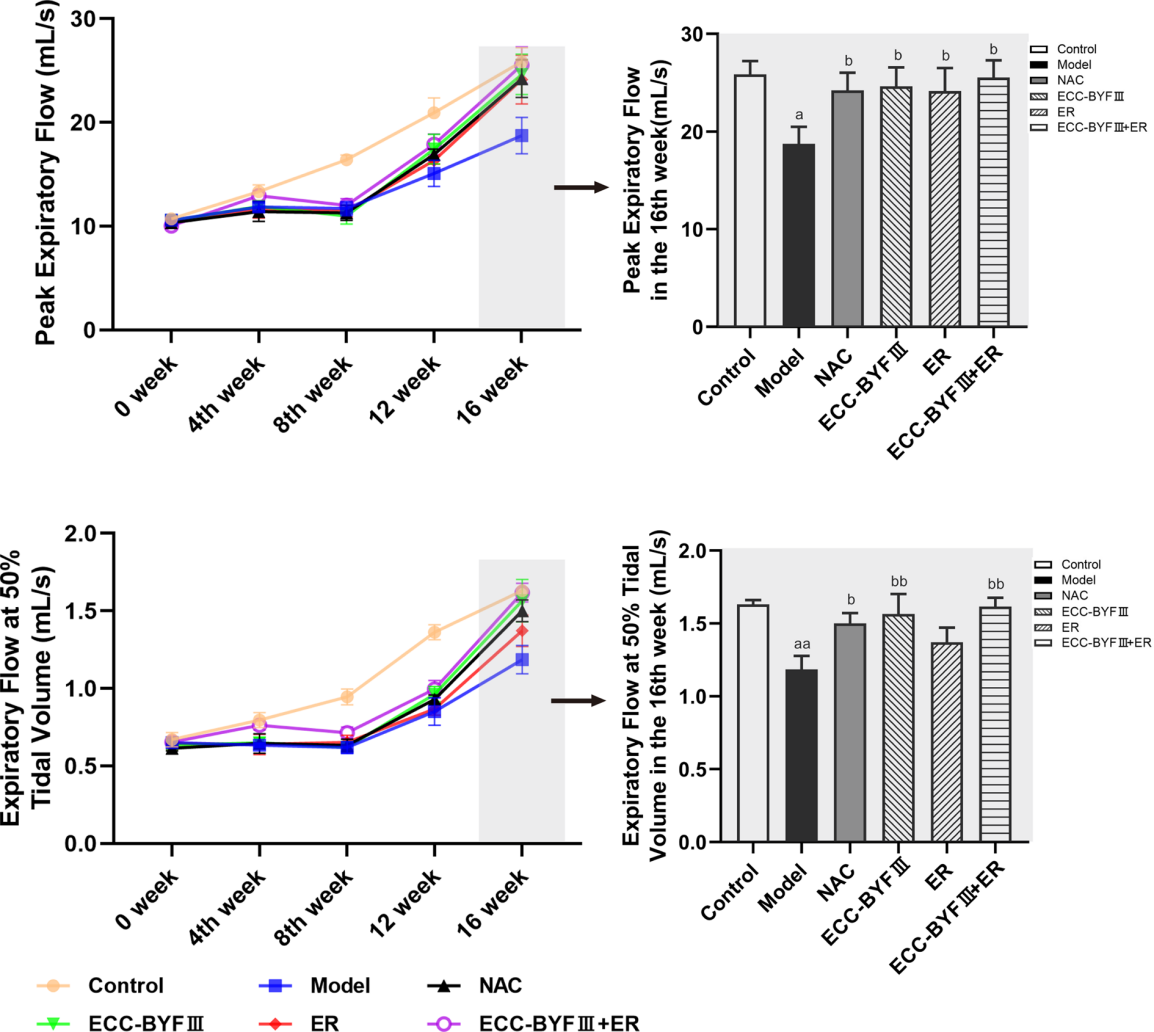
Pulmonary function changes (PEF and EF50) in all groups The values are expressed as mean ± SE, (*n*=6). ^a^*P*<0.05 vs. the normal control group, ^aa^*P*<0.01 vs. the normal control group, ^b^*P*<0.05 vs. the disease model group, and ^bb^*P*<0.01 vs. the disease model group.

Pulmonary function testing is the main method of measuring airflow restriction and diagnosing COPD. During the experimental period, lung function increased significantly in the normal control group and decreased significantly in the disease model group. Lung function in the disease model group was significantly reduced compared with the normal control group, while lung function recovered significantly in all of the treatment groups. After week 16, PEF and EF50 in disease model group were significantly decreased compared with the disease model group (*P*<0.05 and *P*<0.01, respectively). PEF and EF50 were significantly increased in the ECC-BYF III, ECC-BYF III + ER, and NAC groups (*P*<0.05 and *P*<0.01, respectively). There was a trend toward increased EF50 in the ER group; however, this difference did not reach statistical significance (Figure 2).

### ECC-BYF III and ER ameliorate lung injury in COPD rats

**Figure 3:**
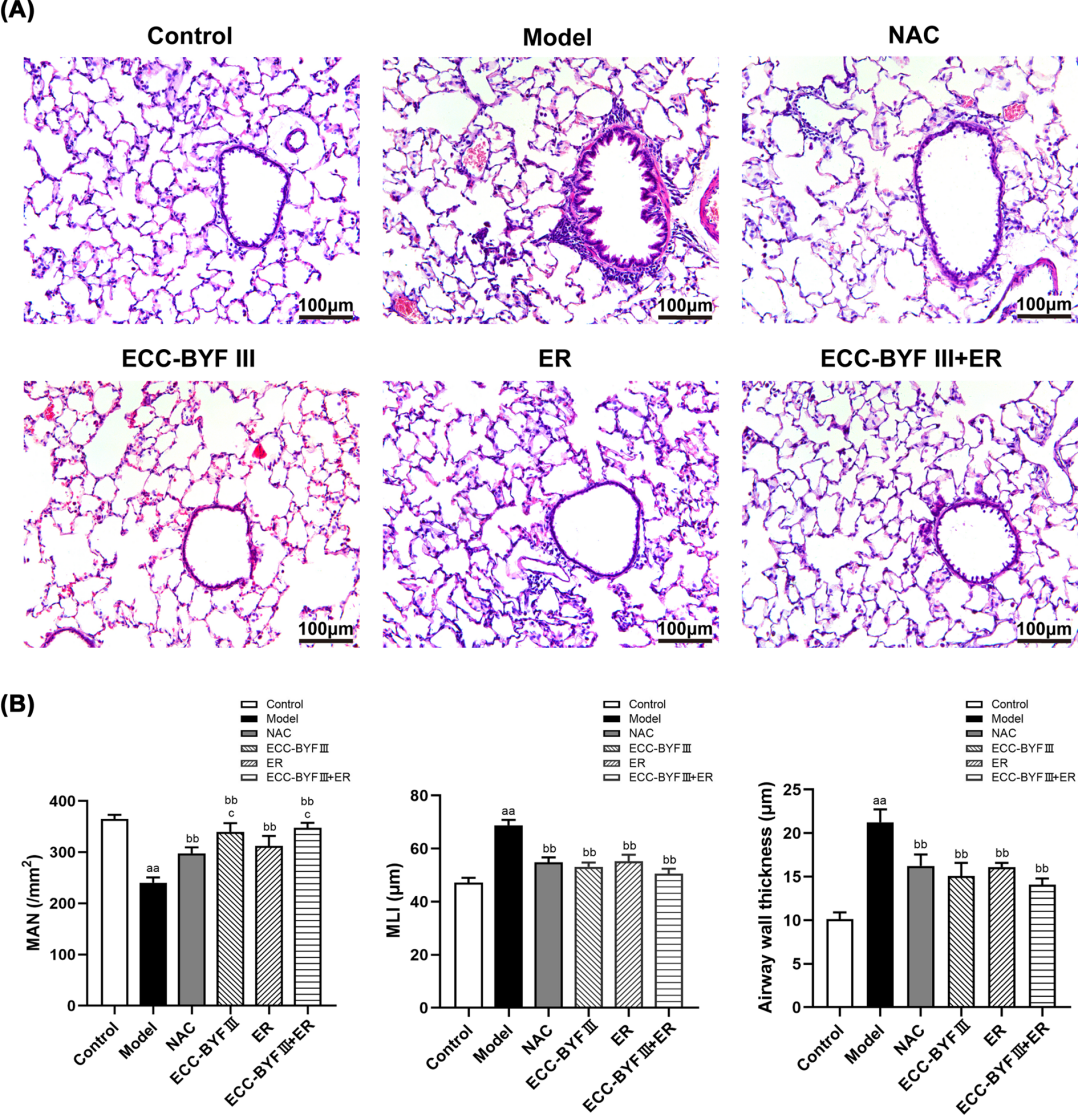
Lung tissue histopathology changes in the lung tissues in all groups (**A**) Photographs of lung tissue histopathology (HE, ×200); (**B**) Changes of MAN, MLI and airway wall thickness. The values are expressed as the mean ± SE, (*n*=6). ^aa^*P*<0.01 vs. the normal control group; ^bb^*P*<0.01 vs. the disease model group; ^c^*P*<0.05 vs. the NAC group.

Histopathological changes are important characteristics of COPD and predominantly manifest as dilation of the alveolar cavities with inflammatory cell infiltration and airway wall thickening. We evaluated histopathological changes in lung tissues from COPD rats. Compared with the normal control group, the lung tissues in disease model group had severe pathological changes including decreased MAN, increased MLI, and increased airway wall thickness (*P*<0.01). These changes were significantly improved in the NAC, ECC-BYF III, ER, and ECC-BYF III + ER (*P*<0.01). This trend was even more pronounced in the ECC-BYF III and ECC-BYF III + ER compared with the NAC group (*P*<0.05, Figure 3).

### ECC-BYF III and ER ameliorated airway inflammation and increases in goblet cell number in COPD rats

**Figure 4:**
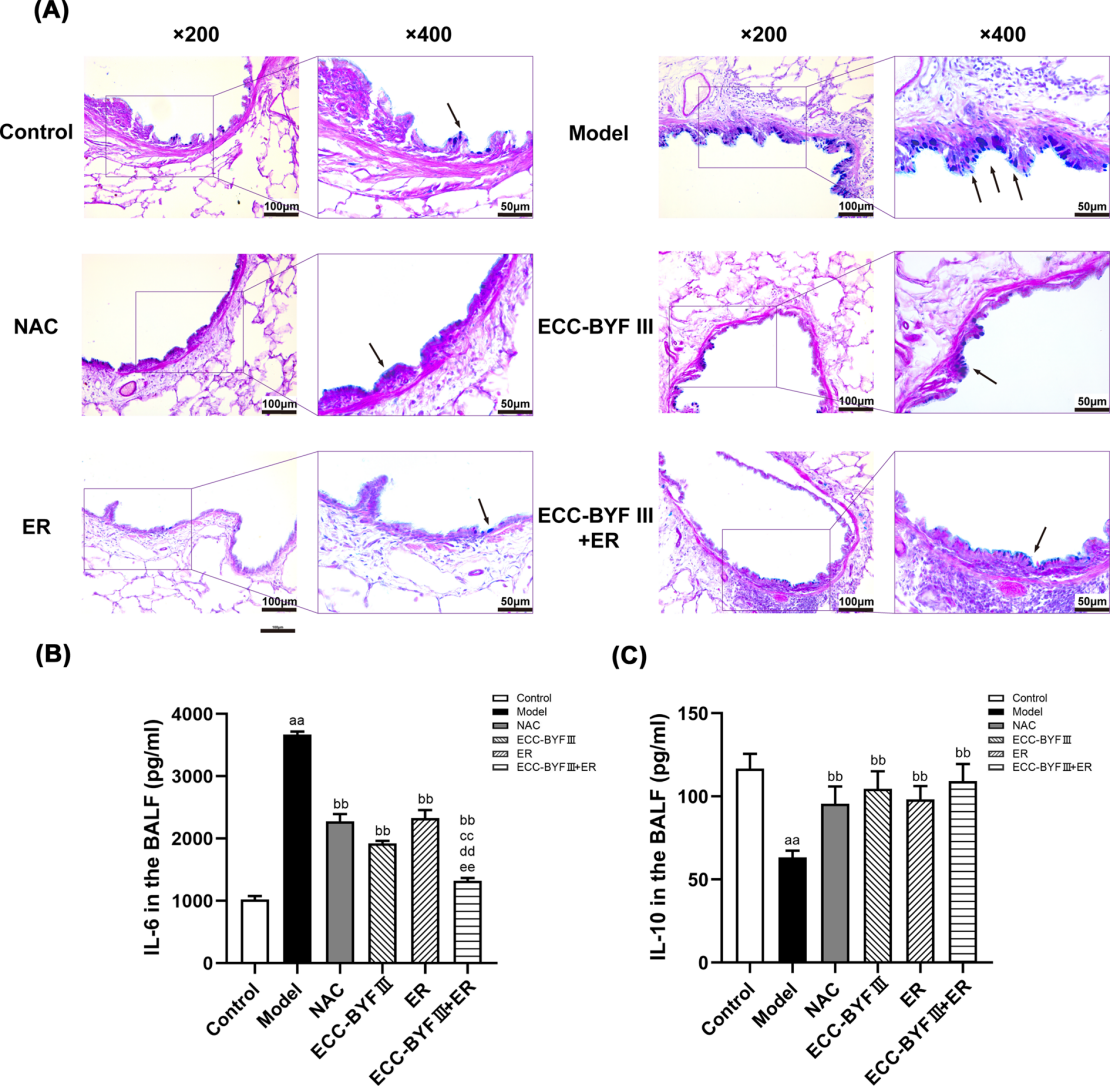
Changes of goblet cells in airway and changes of IL-6 and IL-10 in all groups (**A**) AB-PAS staining photos of the airway in all groups (AB-PAS; ×200, ×400).→: goblet cells. (**B**) Changes of IL-6 in all groups. The data are expressed as the mean ± SE, (*n*=6). ^aa^*P*<0.01 vs. the normal control group; ^bb^*P*<0.01 vs. the disease model group; ^cc^*P*<0.01 vs. the NAC group; ^dd^*P*<0.01 vs. the ECC-BYF III group; ^ee^*P*<0.01 vs. the ER group. (**C**) Changes of IL-10 in all groups. The data are expressed as the mean ± SE (*n*=6). ^aa^*P*<0.01 vs. the normal control group; ^bb^*P*<0.01 vs. the disease model group.

We examined lung and bronchus tissue morphology and airway mucin expression to determine the effects of ECC-BYF III and ER on AMH in COPD rats. Harmful substances such as CS repeatedly stimulate the airways causing an inflammatory response, excessive hyperplasia of goblet cells and mucous glands, and airway epithelial cell damage leading to mucus hypersecretion in COPD. AB-PAS staining demonstrated few blue-stained goblet cells in the airway epithelium of rats from the normal control group and numerous, blue-stained goblet cells in the disease model group. The treatment groups had fewer goblet cells compared with the disease model group, which had higher numbers induced by CS and bacterial inoculation (Figure 4A).

Chronic respiratory inflammation is one of the major pathological features of COPD. To observe the suppressive effects of ECC-BYF III, ER, and ECC-BYF III + ER on airway inflammation in COPD rats, we measured the levels of representative inflammatory factors (IL-6 and IL-10) using ELISA. The ECC-BYF III, ER, ECC-BYF III + ER, and NAC groups had lowers IL-6 levels and higher IL-10 levels in BALF compared with the disease model group (*P*<0.01), which indicated ECC-BYF III, ER, the combination of ECC-BYF III and ER, and NAC inhibited airway inflammation in COPD rats. The ECC-BYF III + ER group had greater suppression of IL-6 levels in BALF compared with other treatment groups (*P*<0.01; Figure 4B,C).

### ECC-BYF III and ER ameliorate mucoprotein levels in lung tissues from COPD rats

**Figure 5:**
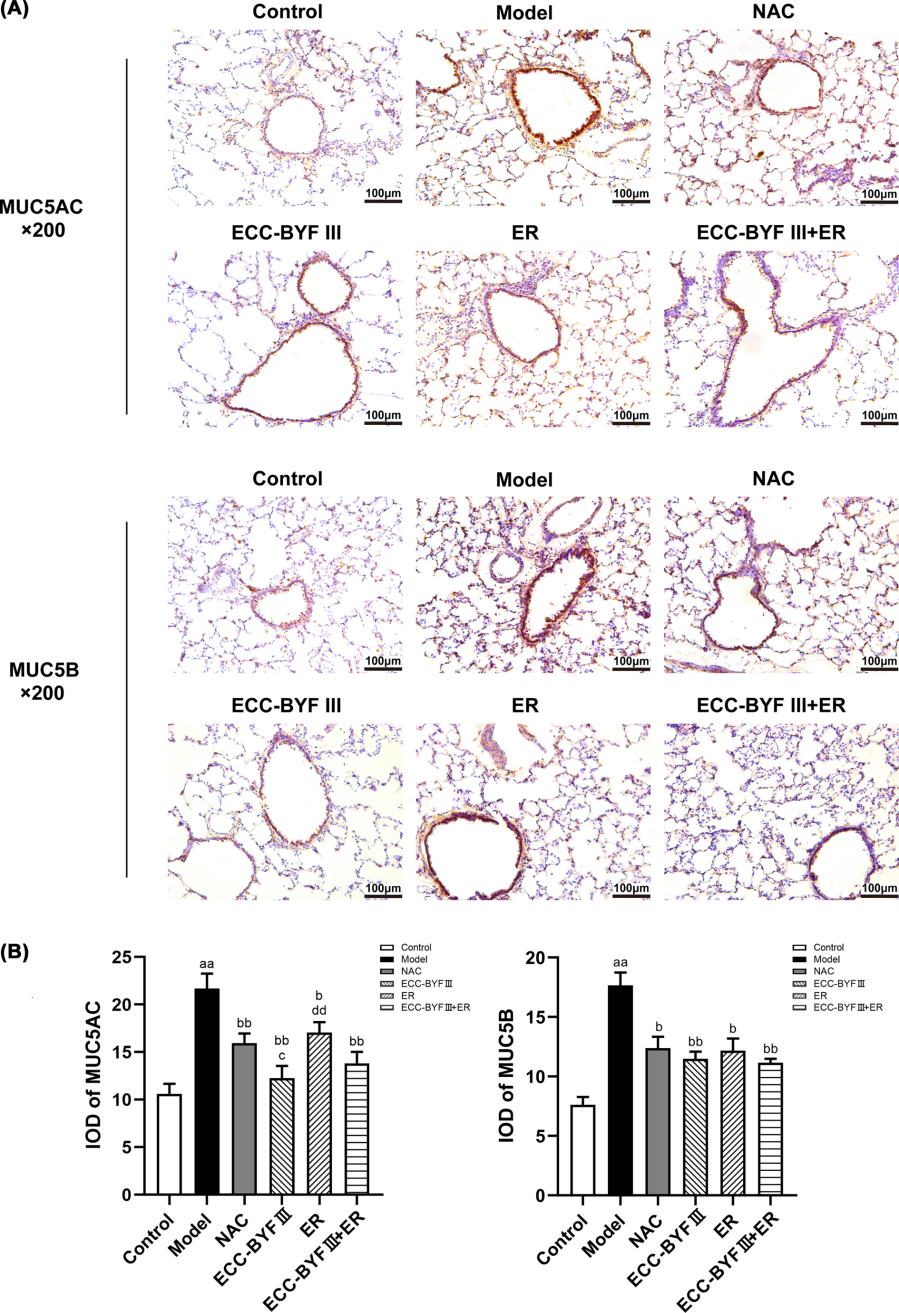
Expression of MUC5AC and MUC5B in the airway (**A**) The expression level of MUC5AC and MUC5B in the airway tested using immunohistochemistry (×200). (**B**) Values are the mean ± SE, (*n*=8). ^aa^*P*<0.01 vs. the normal control group; ^b^*P*<0.05 vs. the disease model group, ^bb^*P*<0.01 vs. the disease model group; ^c^*P*<0.05 vs. the NAC group; ^dd^*P*<0.01 vs. the ECC-BYF III group.

As the major macromolecular component, MUC5AC and MUC5B are predominantly responsible for the biophysical properties of airway mucus. Immunohistochemistry demonstrated that rats in the disease model group had increased MUC5AC and MUC5B levels in lung tissues compared with the normal control group (*P*<0.01). MUC5AC and MUC5B levels were ameliorated in the treatment groups (*P*<0.05, *P*<0.01). MUC5AC levels were significantly lower in the ECC-BYF III group compared with the ER and NAC groups (*P*<0.05 and *P*<0.01, respectively, Figure 5).

### ECC-BYF III and ER ameliorate increased AQP-5 levels in lung tissues from COPD rats

**Figure 6:**
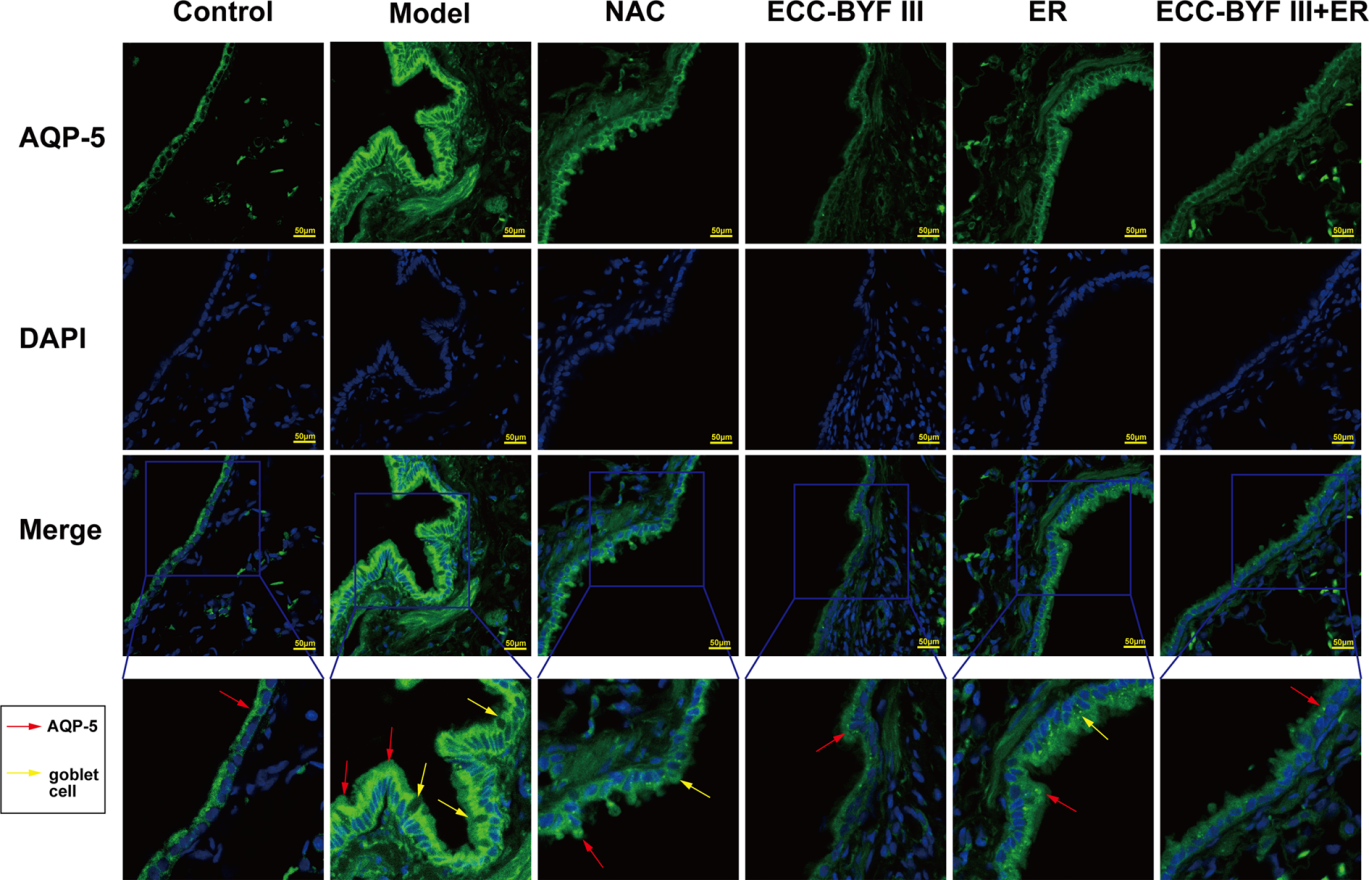
Expression of AQP-5 in the airway The expression level of AQP-5 in the airway tested using immunofluorescence (×400).

Excessive mucin synthesis causes airway inflammation which stimulates goblet cell proliferation and induces AMH [24]. In addition to increases in the absolute amount of mucin, COPD mucus hypersecretion also results in an imbalance in the ratio of mucin to water and salt ratio, with aquaporin 5 (AQP-5) known to regulate the mucin/water–salt ratio. Immunofluorescence demonstrated that rats in disease model group had increased AQP-5 levels in lung tissue compared with the normal control group, with numerous goblet cells observed. The increased AQP-5 levels were ameliorated in the treatment groups (Figure 6).

### ECC-BYF III and ER inhibit AMH in COPD rats by regulating the EGFR/EGFR signaling pathway

**Figure 7:**
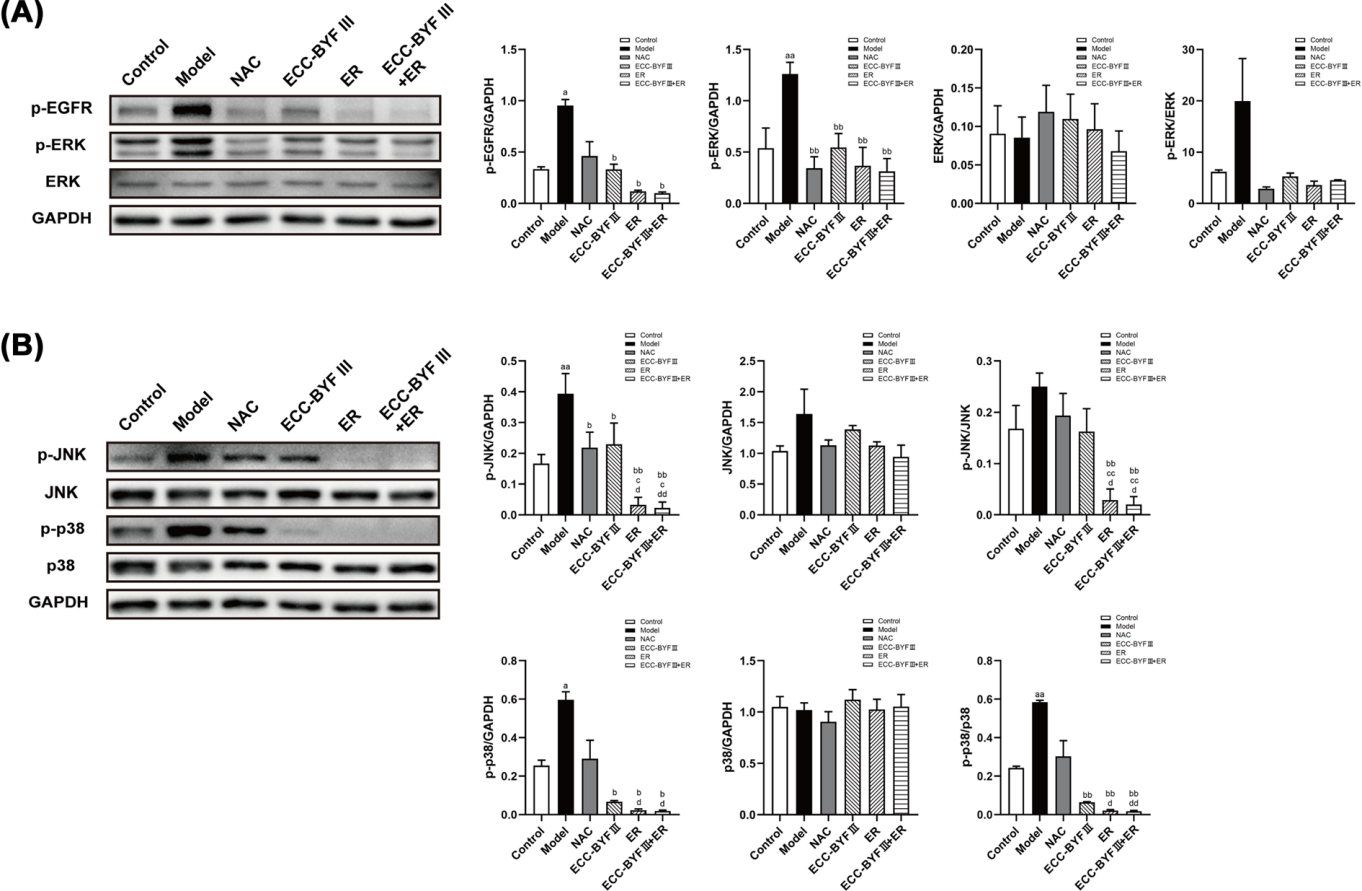
Protein expression of EGFR, ERK, JNK, and p38 in the airway of COPD rats The protein expression of p-EGFR, p-ERK, ERK, p-JNK, JNK, p-p38, and p38. Values are the mean ± SE, (*n*=3). ^a^*P*<0.05 vs. the normal control group; ^aa^*P*<0.01 vs. the normal control group; ^b^*P*<0.05 vs. the disease model group; ^bb^*P*<0.01 vs. the disease model group; ^c^*P*<0.05 vs. the NAC group; ^cc^*P*<0.01 vs. the NAC group; ^d^*P*<0.05 vs. the ECC-BYF III group; ^dd^*P*<0.01 vs. the ECC-BYF III group.

EGFR can activate multiple downstream pathways, including MAPK, which promote goblet cell proliferation and AMH. To further investigate, the potential effects of ECC-BYF III and ER on AMH in COPD rats, we examined the levels of proteins related to the EGFR/MAPK signaling pathway. Western blotting demonstrated that protein levels of phosphorylated EGFR, ERK, JNK, and p38 in the rats from the disease model group were increased compared with the normal control group (*P*<0.05, *P*<0.01), with significantly lower levels in the ECC-BYF III, ER, and ECC-BYF III + ER groups compared with the normal control group (*P*<0.05, *P*<0.01). p-JNK and p-JNK/JNK levels were higher in the ER and ECC-BYF III + ER groups compared with the NAC and ECC-BYF III groups (*P*<0.05, *P*<0.01). p-p38 and p-p38/p38 levels were higher in the ER and ECC-BYF III + ER groups compared with the NAC group (*P*<0.05). There were trends toward increased levels of phosphorylated EGFR and p38 in the disease model groups and trends toward decreased levels of phosphorylated EGFR and p38 in the NAC group; however, there differences did not reach statistical significance. There was no significant change in protein levels of ERK, JNK, or p38 between groups. These findings indicate that EGFR/MAPK signaling may affect AMH through changes in protein phosphorylation (Figure 7).

## Discussion

COPD is a major disease with substantial effects on human health. In recent years, TCM for the treatment of COPD has received increasing attention due to its efficacy and safety. As TCM has been shown to be an effective and safe treatment for lung distention (Feizhang disease), TCM may also represent a treatment option for COPD. Previous multi-center clinical studies have demonstrated that BYF has positive effects in patients with stable COPD by ameliorating clinical symptoms, reducing the frequency of acute episodes, and improving athletic ability and quality of life [11]. Further, BHY was found to be effective in treating COPD rats by enhancing pulmonary function, reducing oxidative stress and inflammation, and regulating protease–antiprotease imbalance [12]. Based on these previous studies of BYF, we screened for the active components of BYF using systematic pharmacology and cell models with repeated *in vitro* and *in vivo* experiments, with the active component identified and termed ECC-BYF I [13]. We then obtained ECC-BYF II by evaluating the therapeutic effects of ECC-BYF I [14]. After further component reduction, we identified five effective components and named them ECC-BYF III. ECC-BYF III can improve AMH; however, the mechanisms underlying this effect remain unclear [16]. Recent studies have demonstrated the positive effects of ER in clinic settings. Reasonable exercise training has positive effects on patients with COPD by improving pulmonary function, enhancing skeletal muscle stamina, improving quality of life, and reducing the cost of treatment [19,25]. Previous studies have shown that ER can improve clinical symptoms, 6-min walking distance, and quality of life in patients with COPD [26]. ECC-BYF III + ER have also been shown to improve pulmonary function and pathological injury in addition to regulating inflammation and immunity in COPD rats [27].

The pathogenesis of COPD is complex. AMH is an independent risk factor for COPD and is associated with disease progression and unfavorable prognosis [14]. AMH has been shown to result in airflow restriction and progressive decline in lung function [28]. The findings of the present study indicates that NAC, ECC-BYF III, and the combination of ECC-BYF III and ER can improve lung function and lung injury, with ECC-BYF III and the combination of ECC-BYF III and ER having greater efficacy in improving MAN than NAC. Goblet cell metaplasia and increased mucin production are important manifestations of AMH. In the present study, we observed increased numbers of goblet cells and increased protein levels of MUC5AC, MUC5B, and AQP-5 in COPD rats. Further, reduced goblet cell numbers and decreased MUC5AC, MUC5B, and AQP-5 levels were observed in rats treated with NAC, ECC-BYF III, ER, or a combination of ECC-BYF III and ER. ECC-BYF III had a greater effect on MUC5AC levels than ER and NAC. Airway inflammation is promoted by external hazardous substances and causes proliferation of goblet cells and mucous glands, with damage to airway epithelial cells contributing to AMH [29]. To further explore the mechanisms underlying the pathogenesis of AMH, we measured inflammatory markers in BALF and demonstrated increased IL-6 levels and decreased IL-10 levels in COPD rats. All treatments evaluated in the present study affected the levels of inflammatory markers in BALF, with the combination of ECC-BYF III and ER found to have a greater suppressive effect on IL-6 levels than other treatments.

Goblet cell hyperplasia and impairment of mucociliary scavenging are the major mechanisms underlying the pathogenesis of AMH, with both regulated by multiple signaling pathways including the EGFR/MAPK pathway. In the present study, we observed significantly decreased levels of phosphorylated EGFR and significant increased levels of phosphorylated p38, ERK, and JNK in the disease model group indicating that the EGFR/MAPK pathway is activated in COPD rats. Interestingly, ECC-BYF III, ER, and the combination of ECC-BYF III and ER reduced the protein levels of phosphorylated EGFR, ERK, JNK, and p38, with the combination of ECC-BYF III and ER have greater effect than ECC-BYF III and NAC in decreasing the levels of p-JNK and p-p38. Accordingly, ECC-BYF III, ER, and the combination of ECC-BYF III and ER may improve AMH in COPD rats through inhibiting EGFR and downstream MAPK pathways including ERK, JNK, and p38 signaling. Further, the combination of ECC-BYF III and ER having greater effects on suppressing levels of phosphorylated JNK and p38 protein.

In conclusion, ECC-BYF III, ER, and the combination of ECC-BYF III and ER were found to have efficacy in treating AMH in COPD rats. Each treatment resulted in improved lung function and lung tissue injury, reduced inflammatory cell infiltration, reduced goblet cell number, and decreased levels of MUC5AC and MUC5B in lung tissues. ECC-BYF III and the combination of ECC-BYF III and ER had greater effects on improving MAN, with ECC-BYF III treatment leading to higher levels of MUC5AC in BALF compared with treatment with ER. The combination of ECC-BYF III and ER had the greatest suppressive effect on IL-6 compared with other treatments. The effects of ECC-BYF III and ER on AMH in COPD rats may be mediated by inhibition of the EGFR/MAPK signaling pathway.

## Supplementary Material

Supplementary Material S1

## Data Availability

Data for the present study can be obtained from the corresponding author according to the rules.

## Abbreviations

AB-PAS: Alcian Blue/periodic acid-Schiff
AMH: airway mucus hypersecretion
AQP-5: aquaporin 5
BALF: bronchoalveolar lavage fluid
COPD: chronic obstructive pulmonary disease
CS: cigarette smoke
ECC-BYF I: effective-component compatibility of Bufei Yishen formula I
ECC-BYF II: effective-component compatibility of Bufei Yishen formula II
ER: exercise rehabilitation
GOLD: Global Initiative for Chronic Obstructive Lung Disease
KP: Klebsiella pneumoniae
MAN: mean alveolar number
MLI: mean linear intercept
NAC: acetylcysteine
PEF: peak expiratory flow
p-EGFR: phosphorylated EGFR
p-ERK: phosphorylated ERK
p-JNK: phosphorylated JNK
SD: Sprague–Dawley
TCM: Traditional Chinese Medicine
