# Supplementary material for: Effective-compound combination of Bufei Yishen formula III combined with ER suppress airway mucus hypersecretion in COPD rats: via EGFR/MAPK signaling
Source: Biosci Rep. 2023 Nov 10;43(11):BSR20222669. doi: 10.1042/BSR20222669 (PMC10643050; doi:10.1042/BSR20222669)
Supplement: Supplementary Material S1 [file BSR-2022-2669_supp.pdf]

Supplementary material 3: The diagram of rats running on the experimental treadmill.

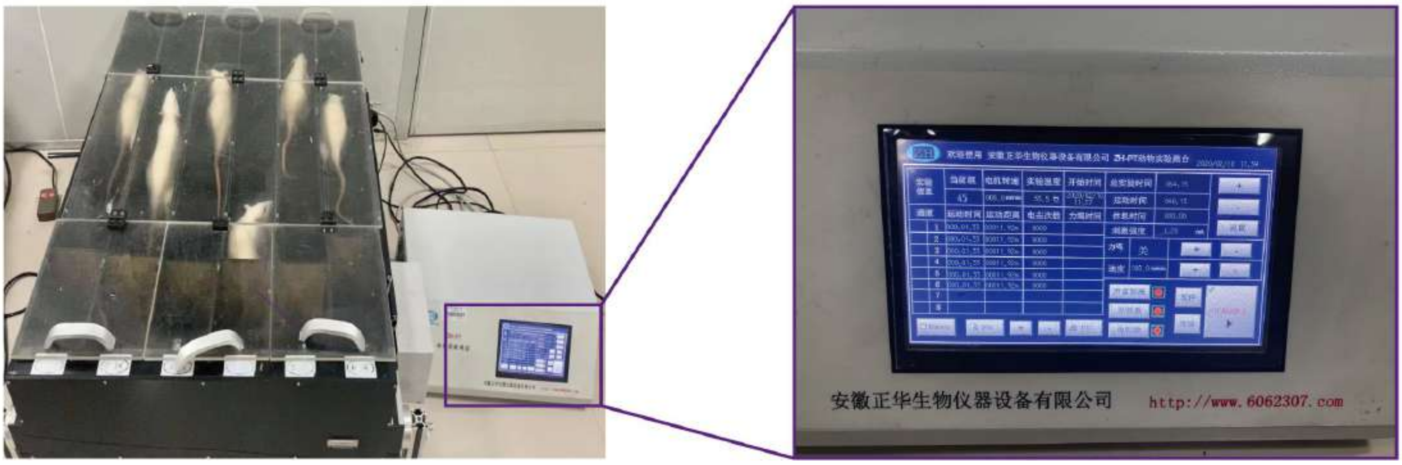

Supplementary material 1. Determine the speed of exercise

Speed	Status of rats	Evaluation
6m/min	Stuck at the end of the conveyor belt	infeasible
8m/min	Go at a canter	feasible
10m/min	Moving too fast and will causing injury in rats	infeasible

Supplementary material 2. Determine the time of exercise

Time	Group A	Group B	Group C	Evaluation
10min	The rats were in good condition	-	-	feasible
20min	-	The rats were in good condition	-	feasible
30min	-	-	The rats had poor spirit and little activity	infeasible
